# Tamoxifen inhibits histidine kinases of *M. tuberculosis* two-component signaling systems

**DOI:** 10.1128/spectrum.01880-25

**Published:** 2025-12-16

**Authors:** Abhishek Garg, Devendra Pratap Singh, Mansi Pandit, Vandana Malhotra, Deepak Kumar Saini

**Affiliations:** 1 Department of Biochemistry, University of Delhi South Campus 93081, New Delhi, India; 2 Department of Biochemistry, Sri Venkateswara College, University of Delhi 208742 , New Delhi, India; 3 Department of Developmental Biology and Genetics, Indian Institute of Science 29120, Bengaluru, Karnataka, India; 4 Bioinformatics Infrastructure Facility, Sri Venkateswara College, University of Delhi 208742 , New Delhi, India; 5 Department of Bioengineering, Indian Institute of Science 29120, Bengaluru, Karnataka, India; 6 Centre for Infectious Disease Research, Indian Institute of Science 29120, Bengaluru, Karnataka, India; University at Albany, Albany, New York, USA

**Keywords:** *M. tuberculosis*, two-component signaling system, histidine kinase, tamoxifen, PhoR and MtrB, small-molecule inhibitors, anti-TB drug target

## Abstract

Two-component signaling systems (TCSSs) serve as primary signal transduction pathways in bacteria, regulating essential processes including growth, physiology, and virulence, making them attractive drug targets. In *Mycobacterium tuberculosis* (*M. tb*), the PhoPR system plays a critical role in pathogenesis, with the PhoR histidine kinase (HK) functioning at the intersection of cognate and noncognate signaling networks. Using PhoR as a prototypical HK, we hypothesized that targeting this system would compromise *M. tb’s* adaptive capacity. We developed and optimized a high-throughput screen of pharmacologically active small-molecule libraries to identify PhoR autophosphorylation inhibitors. Selected compounds were evaluated using kinase assays, protein interaction analyses, molecular docking studies, and growth inhibition using *Mycobacterium bovis* BCG. Screening identified 11 potential inhibitors, with tamoxifen (TAM) demonstrating the most potent activity. TAM inhibited PhoR autophosphorylation at micromolar concentrations both *in vitro* and *in vivo*. Mechanistic studies revealed that TAM competitively binds to the ATP-binding pocket of PhoR with a dissociation constant (K_d_) of 108.5 ± 44 nM. Although maximum inhibition was found with PhoR, additional screening of HKs revealed MtrB as another low-affinity target of TAM (K_d_ = 412 ± 83 nM). Treatment with TAM significantly inhibited *M. bovis* BCG growth in culture and suppressed PhoPR-regulated acid-responsive gene expression. Our findings establish PhoR HK as a promising antimycobacterial drug target and demonstrate the potential for repurposing the clinically approved anticancer drug TAM as an anti-tuberculosis therapeutic with TCS-targeting effects. This work provides proof of concept for targeting bacterial TCSSs and supports further development of TAM derivatives for tuberculosis treatment.

**IMPORTANCE:**

Two-component signaling systems are essential for bacterial growth, metabolism, and survival, making them ideal candidates for selective antimicrobial therapy. Tamoxifen (TAM), a well-known anticancer drug, has recently been shown to exhibit antimicrobial activity and is emerging as a potential anti-tuberculosis (TB) agent. In this study, we report for the first time that TAM inhibits *Mycobacterium tuberculosis* histidine kinases, PhoR and MtrB, implicated in virulence. Using a combination of biochemical and computational biology techniques, we demonstrate that TAM competes with ATP for PhoR binding and impairs its autophosphorylation activity, thereby disrupting downstream regulation of gene expression. Dissociation kinetics revealed that in comparison to PhoR, TAM bound MtrB with a lower affinity. These findings establish PhoR as a novel drug target, highlight a plausible mechanism of TAM’s antimycobacterial action, and, more importantly, support its repurposing as a promising therapeutic candidate against TB.

## INTRODUCTION

Tuberculosis (TB) remains a leading cause of death globally (1). The disease is caused by *Mycobacterium tuberculosis (M. tb*), a pathogen that has evolved sophisticated mechanisms to survive within infected hosts. Upon infection, *M. tb* can follow two distinct pathways: it may cause immediate active disease or enter a dormant, asymptomatic state known as latent TB infection (2). Exposure to host immune responses directs significant changes in mycobacterial gene expression that allow it to transition from active growth to a slow-growing, nonreplicative state (3, 4). This dormancy mechanism enables the bacteria to survive undetected within the host for decades, serving as a reservoir for future disease reactivation.

Signal transduction systems are central to this adaptability and are vital in regulating mycobacterial pathogenesis, persistence, and virulence (5). One such family of signaling systems is the two-component signaling system (TCSS), which is responsible for sensing environmental cues and executing widespread changes in gene expression patterns *(reviewed in* [6, 7]). Typically, a TCSS consists of a *sensory protein,* usually a histidine kinase (HK), which senses the environmental stimulus and undergoes autophosphorylation on a conserved histidine residue. This phosphate is then transferred to a conserved aspartate residue on a *regulatory protein* called the response regulator (RR), a DNA-binding transcription factor capable of regulating the transcription of downstream regulons (8).

*M. tuberculosis* has 12 paired two-component systems, six orphan regulators, and two orphan HKs (6, 9). Among them, RRs MtrA and PrrA are essential for *M. tb* survival (10, 11), and PhoPR and DevRS systems are implicated in mycobacterial latency and virulence (5, 12–15). The PhoPR TCSS consists of a membrane-bound HK PhoR that is responsive to low pH and activates a signaling cascade by phosphorylating its cognate RR PhoP, which regulates transcription of its downstream regulon (16–19). The PhoPR regulon is known to be responsible for regulating the synthesis of complex cell wall lipids such as di-acyltrehaloses and poly-acyltrehaloses essential for virulence (15, 20–24), for regulation of the enduring hypoxic response and respiration, secretion of virulence factors (25), and most importantly for arresting the maturation of phagosomes, an event that is crucial for the intracellular survival of mycobacteria (26). In fact, a single point mutation in the *phoP* gene contributes significantly to the attenuation of *M. tb* H37Ra, the isogenic counterpart of the virulent *M. tb* H37Rv laboratory strain (24, 27, 28). Studies have demonstrated improved clearance of H37Ra as compared to H37Rv in infected alveolar macrophages and murine models, possibly due to the inability of H37Ra to arrest the movement of lysosomes in the outer periphery of the cell, resulting in increased localization toward the nucleus, causing mycobacterial cell death (25, 27). Further, Abramovitch et al. have reported an acid and phagosome regulator (*apr*) locus activated by PhoPR under an acidic pH environment to secrete molecules/peptides that inhibit phagosome maturation and prevent killing of the pathogen (16). Notably, an *M. tb phoPR* knockout strain is attenuated for intracellular growth in human and mouse models of infection (23, 24) and is a component of MTBVAC, the first live-attenuated *M. tb-*based vaccine currently in clinical trials (29).

This study explored HKs as unique targets for developing novel antitubercular drugs. We targeted PhoR autokinase activity as a template, screened small-molecule inhibitors using a high-throughput assay, and identified the anticancer drug, tamoxifen (TAM), as a lead inhibitor. Biophysical, biochemical, and computational tools helped characterize the TAM-HK interaction, facilitating validation of its inhibitory effect on mycobacterial growth and providing a plausible mechanism of action. The potential applicability of TAM as a repurposed anti-TB drug is discussed.

## RESULTS

### Development of a high-throughput screening (HTS) assay for PhoR autophosphorylation inhibitors

The PhoPR TCS is implicated in the regulation of mycobacterial virulence, modeling cell wall lipids, and driving metabolic adaptation during infection (21, 23, 24, 28, 30, 31). Further, its network connecting the cognate and multiple noncognate signaling pathways posits a good rationale to investigate PhoR HK as a potential target for the development of antitubercular drugs (14, 31–35).

**Fig 1:**
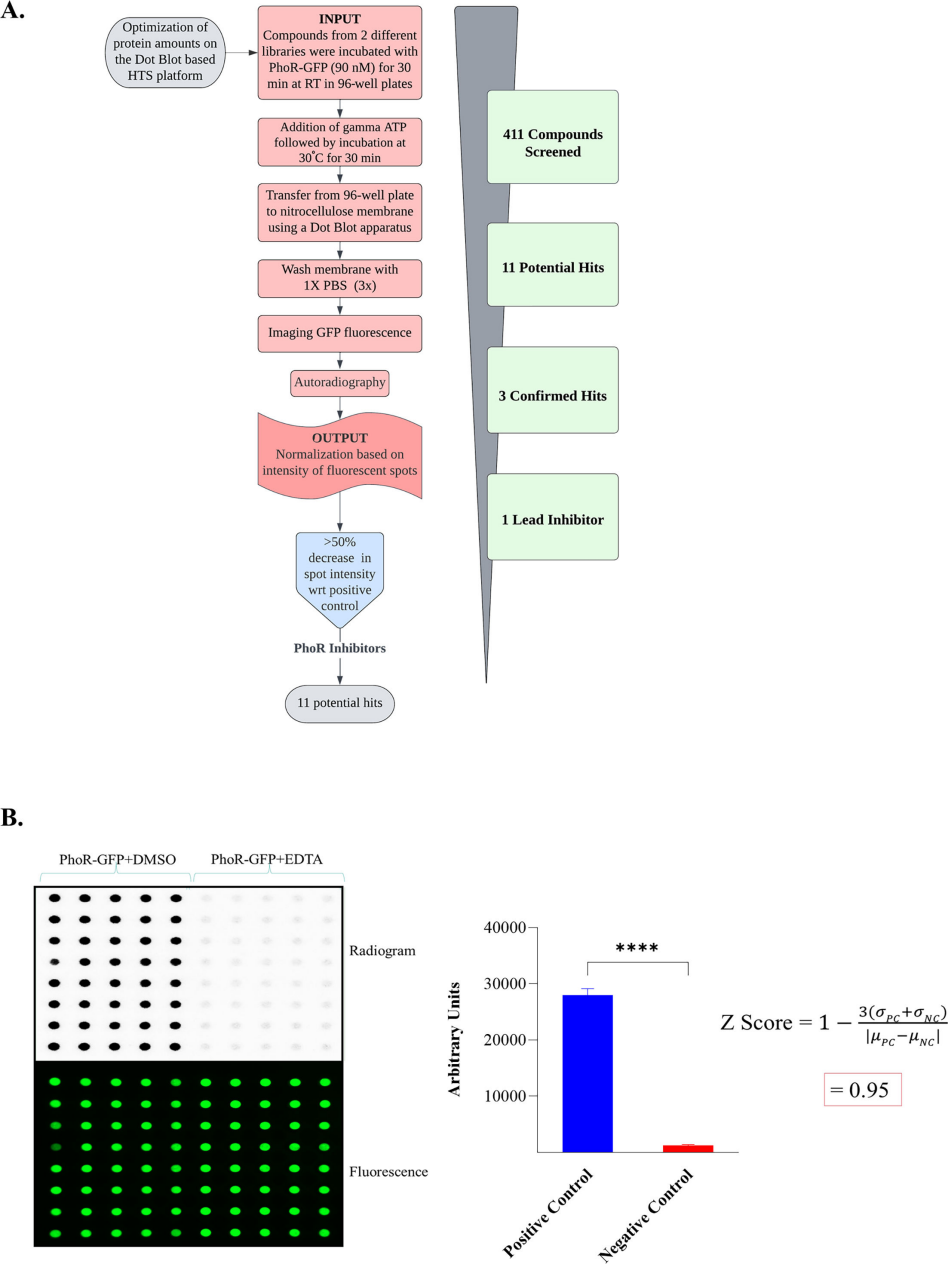
Development of an HTS assay for PhoR autokinase inhibitors. (**A**) Schematic of the workflow for developing the HTS platform for PhoR-GFP autophosphorylation inhibition. (**B**) *Left panel*, autoradiogram of PhoR-GFP autophosphorylation (top) normalized with the corresponding fluorescence intensity (bottom). *Right panel*, densitometric analysis of the radiogram to calculate the Z-score of the assay. PhoR-GFP + EDTA and PhoR-GFP + DMSO were NC and PC, respectively. **** denotes *P* < 0.0001.

Towards this, we developed a high-throughput 96-well plate assay to screen for compounds that inhibit PhoR autophosphorylation. We optimized an HTS platform targeting PhoR autokinase activity. The schematic workflow of the HTS assay is shown in Fig. 1A. Autophosphorylation of PhoR-GFP was scored by measuring the intensity of spots on the autoradiogram (Fig. 1B). The Z-score of the radioactive dot blot assay developed was calculated to be 0.95, indicating high-performance quality of the assay, a prerequisite for identification of an effective inhibitor (Fig. 1B, *right*). In this experiment, we used GFP-tagged PhoR to normalize the protein amounts to account for well-to-well variation. Reactions in the presence of EDTA and dimethyl sulfonyl oxide (DMSO) served as negative (NC) and positive controls (PC), respectively (Fig. 1B). The assay was validated multiple times and proved to be fast, sensitive, and reproducible.

### Pilot screening of compounds and validation of hits

**Fig 2:**
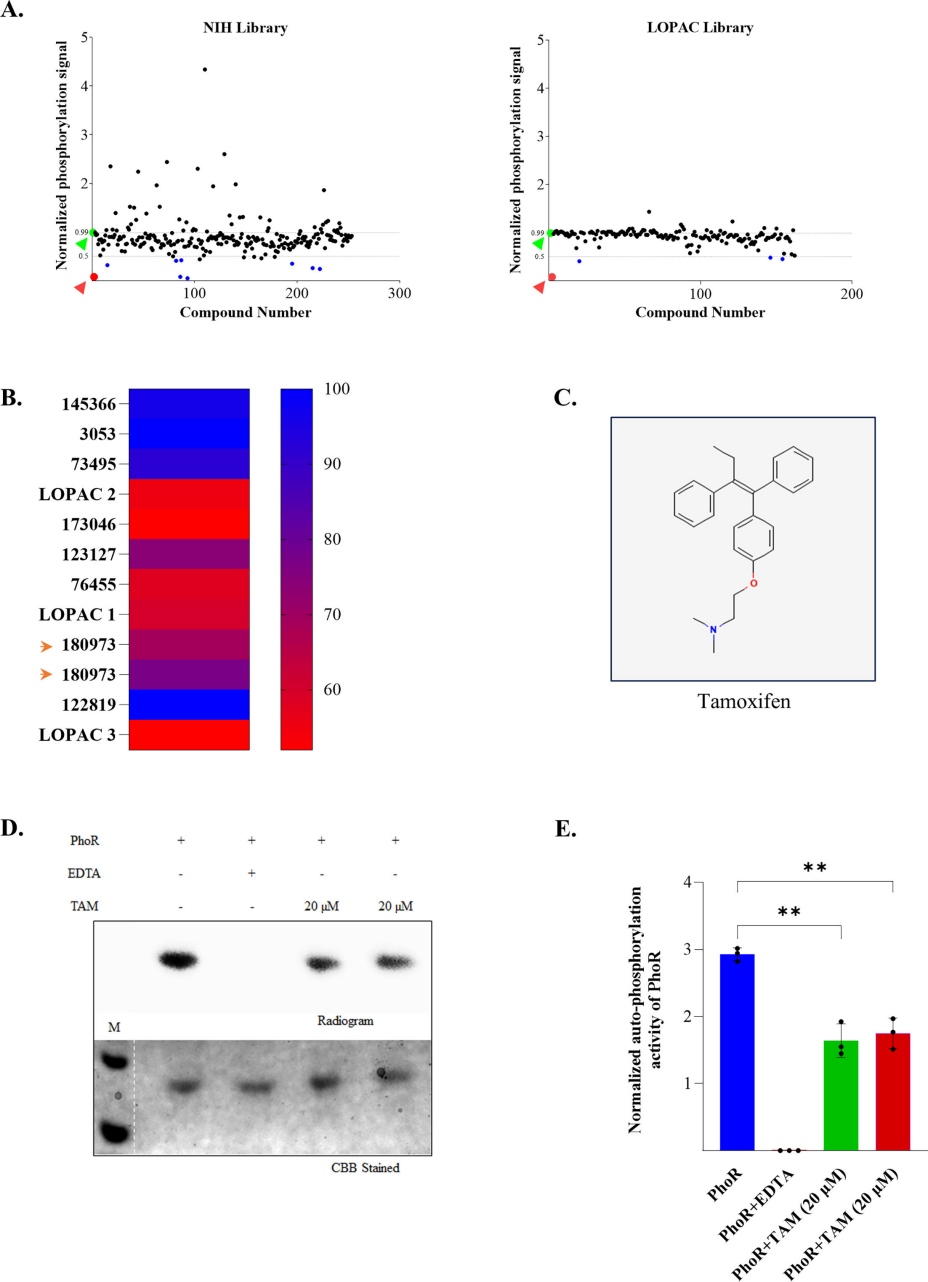
Screening and identification of compounds that inhibit PhoR autokinase activity. (**A**) Plot of PhoR autophosphorylation signal intensity normalized with the amount of protein against the compound number. *Left panel*, screening of compounds from the NIH Library (*n* = 230 compounds); *Right panel*, from the LOPAC library (*n* = 181 compounds). Red and green dots indicate autophosphorylation in the presence of EDTA (NC) and the absence of any inhibitor (PC), respectively. (**B**) Heat map showing relative inhibitory activity (%) of potential hits, with blue bars indicating greater inhibition. The identity and CAS number of the hits are mentioned in Table 1. Arrowheads indicate TAM (180973). (**C**) Structure of TAM; (PubChem CID 2733526). (**D**) *In vitro* kinase assay of PhoR with TAM. Reaction in the presence of EDTA served as an NC (Lane 3). Assays were performed in two independent experiments in duplicates with PhoR and 20 μM TAM (Lanes 4 and 5), incubated for 60 min, visualized by autoradiography, and one representative image is shown. (**E**) Densitometric analysis of the image in Fig. 2D is shown, highlighting the inhibitory action of TAM. Data are presented as mean ± SD of values obtained from all replicates. ** denotes *P* < 0.01.

Two different chemical compound libraries (NIH-NCATS oncology set and Sigma LOPAC^1204^) were used, and ~500 compounds were screened for inhibitors of PhoR autophosphorylation (Fig. 2A). Screening was done in duplicates, and the readout was obtained by measuring spot intensities (see Materials and Methods section). Compounds inhibiting more than 50% of autophosphorylation *in vitro* were considered as potential PhoR inhibitors (Fig. 2A, *blue dots*). We identified 11 hits, which were taken forward for validation. Their respective percentage inhibition is presented as a heat map (Fig. 2B) with corresponding identities and CAS numbers listed in Table 1.

**TABLE 1:** List of potential hits obtained in the HTS assay targeting PhoR autophosphorylation^*a*^

ID^*b*^	Compound name (CAS no.)^*c*^
145366	2-Propanol, 1,1′-[(1-methylethylidene) bis(4,1- phenyleneoxy)] bis[3-[(1,1,3,3-tetramethylbutyl)amino]-, dihydrochloride
3053	Actinomycin D (50-76-0)
73495	B 132742
LOPAC 2	Caroverine (23465-76-1)
173046	Diphenyllead diacetate (6928-68-3)
123127	Doxorubicin (25316-40-9)
76455	Peliomycin (1404-20-2)
LOPAC 1	Protoporphyrin IX disodium (50865-01-5)
180973	TAM citrate (54965-24-1)^*d*^
180973	TAM citrate (54965-24-1)^*d*^
122819	Teniposide (29767-20-2)
LOPAC 3	Yohimbine (146-48-5)

^*a*^ Shaded rows indicate three confirmed hits obtained in the HTS assay.

^*b*^ Cancer Chemotherapy National Service Center number is a compound identifier assigned by DTP to identify an agent, product, or LOPAC compound.

^*c*^ Identity of compounds and CAS number.

^*d*^ Results from two independent plates.

Colored compounds with absorption spectra coinciding with that of GFP, for example, doxorubicin (with an absorption maximum of 496 nm), were classified as false positives as they yielded very high fluorescence values. Some compounds acted as chelators and were unfavorable for downstream assays, while many were not commercially available for further testing. Only three compounds displayed more than 50% inhibition in the dot blot without interference from any other parameter from the assay (Fig. 2B and Table 1, *shaded*). Coincidentally, two of the three compounds originating from two different libraries were the well-known breast cancer therapeutic, TAM (36) (Fig. 2C) and the other compound was actinomycin D.

We confirmed the inhibitory action of TAM on PhoR kinase activity in two independent experiments. Autophosphorylation of PhoR was inhibited in the presence of 20 μM TAM (Fig. 2D). Densitometric analysis of the replicates revealed an average value of ~56% reduction in PhoR autophosphorylation in the presence of TAM (Fig. 2E). Incidentally, this value is lower than the inhibition observed in the high-throughput format with 10 μM TAM (~73% inhibition) (Fig. 2B). Understandably, this disparity could stem from the differences in the experimental setup, gel vs the plate format. Since actinomycin D is a known carcinogen, further experiments were done with TAM only.

### Binding studies of TAM to PhoR

**Fig 3:**
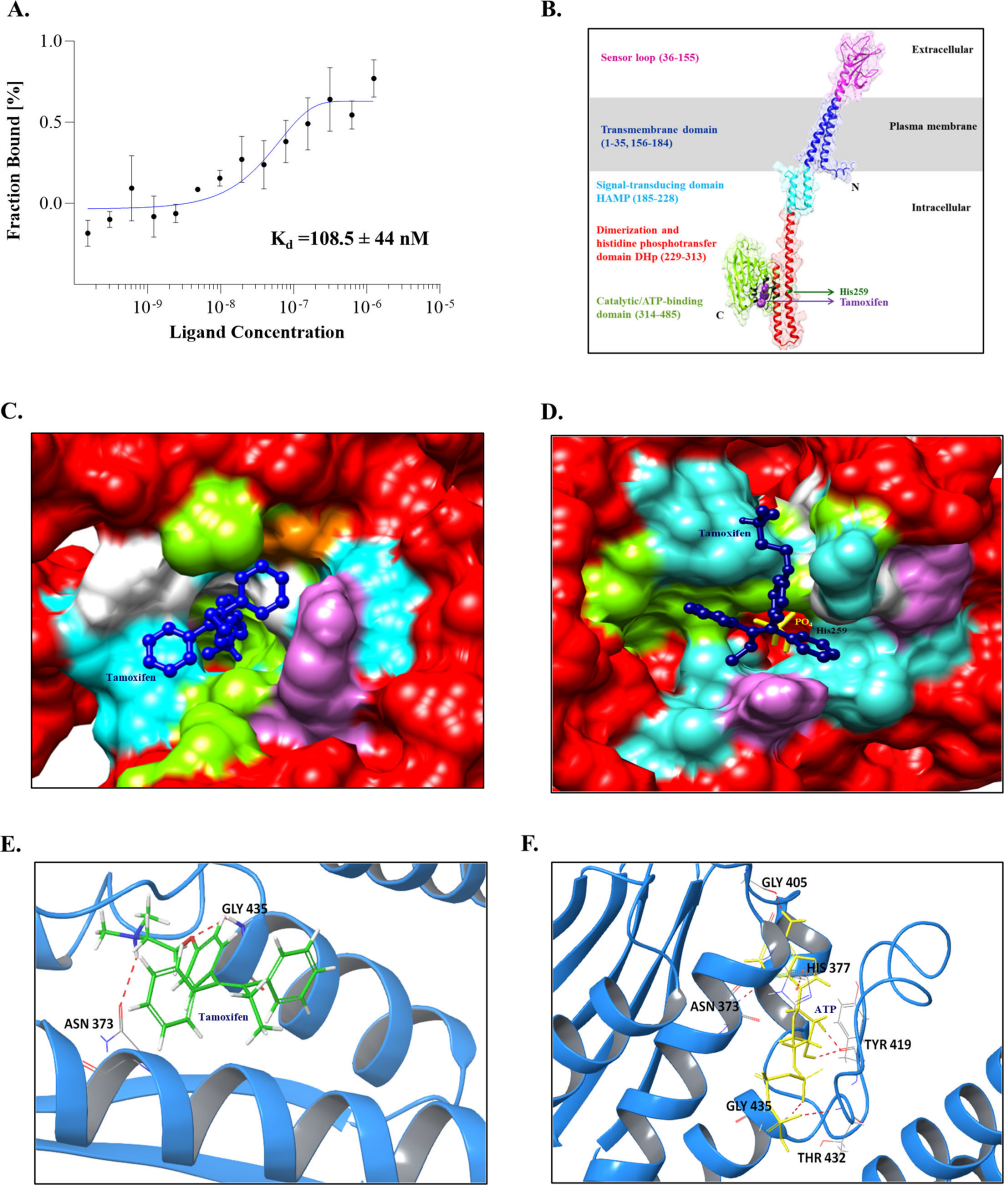
Binding analysis of TAM with PhoR. (**A**) MST showing K_d_-Fit analysis of PhoR-GFP with TAM. The experiment was performed in triplicate, and a representative graph is shown. (**B**) PhoR predicted structure model and binding sites. Ribbon diagram representation of the PhoR model structure obtained from the AlphaFold database, highlighting the domains and amino acid residue numbers, His^259^, and TAM bound at *Site1*. (**C and D**) Surface representation of the kinase domain of PhoR (red) and PhoR~*P* (red with yellow PO_4_^3−^) with TAM (dark blue ball and stick model)-docked complex, respectively, highlighting interacting residues (hydrophobic, green; polar, cyan; positively charged, purple; negatively charged, orange; and glycine, white) and (**E and F**) protein-ligand interactions within 0.5 nm are shown (hydrogen bonds, pink lines) for PhoR-TAM and PhoR-ATP, respectively.

Characterization of the interaction between TAM and PhoR is crucial for evaluating its specificity and efficacy. To achieve this, we utilized microscale thermophoresis (MST) to determine the binding affinity of TAM with PhoR. We observed that TAM binds to PhoR *in vitro* with a dissociation constant (K_d_) of 108.5 ± 44 nM (Fig. 3A).

We used computational tools to gain further insights into the biophysical aspects of inhibitor binding to PhoR. The three-dimensional structure of PhoR was obtained from the AlphaFold database (see the Materials and Methods section). Structure validation statistics revealed that the predicted PhoR structure (485 amino acids) was of very good quality, having an ERRAT quality factor of 94.27% and 96.7% residues in the favorable region of the Ramachandran plot (Fig. S1) (37, 38). Since binding of TAM to PhoR inhibits its autophosphorylation activity, it was essential to understand the structural basis of this observation. PhoR domain architecture (Fig. 3B) along with the surface representation of PhoR-TAM (Fig. 3C), PhoR~P-TAM complexes (Fig. 3D), and the interacting residues of PhoR-TAM complex were generated (Fig. 3E). Binding site analysis with the kinase domain of PhoR spanning amino acid residues 256 to 470, using SiteMap module (39), resulted in three probable TAM-binding sites, i.e., *site 1*, spanning residues 242–445; *site 2*, spanning residues 296–462; and *site 3*, spanning residues 260–431. Molecular docking of PhoR, with TAM at these sites, was performed using the Extra-Precision (XP) mode of the Glide module (40). Results indicate that TAM had the most significant interaction with residues at *site 1*, with a binding free energy value (ΔG) of −52.68 kcal/mol as opposed to −32.55 kcal/mol for *site 2*, and −30.91 kcal/mol for *site 3*. We noted that molecular docking of TAM with PhoR~*P* shifted the free energy change toward the positive side (−1.25 kcal/mol) (Fig. 3D) as compared to when it binds unphosphorylated PhoR (Fig. 3C). These observations highlight the preferential binding of TAM to unphosphorylated PhoR at *site 1* (−52.68 kcal/mol).

Since *site 1* is located very close to the ATP-binding domain of PhoR, we investigated whether it overlaps with TAM binding. Molecular docking analysis revealed an ~80% overlap between the TAM and ATP-binding sites in PhoR at *site 1* (Table S1), suggesting possible competition between the two for binding to PhoR. The amino acid residues of the PhoR kinase domain interacting with TAM and ATP at *site 1* are listed in Table S1. Comparative binding energy analysis revealed that TAM binding to PhoR is favored as it has a higher negative free energy value, unlike that observed with ATP (−20.71 kcal/mol). These results indicate a stable TAM-PhoR complex that precludes ATP binding and results in inhibition of PhoR autophosphorylation.

### Mechanism of action of TAM

**Fig 4:**
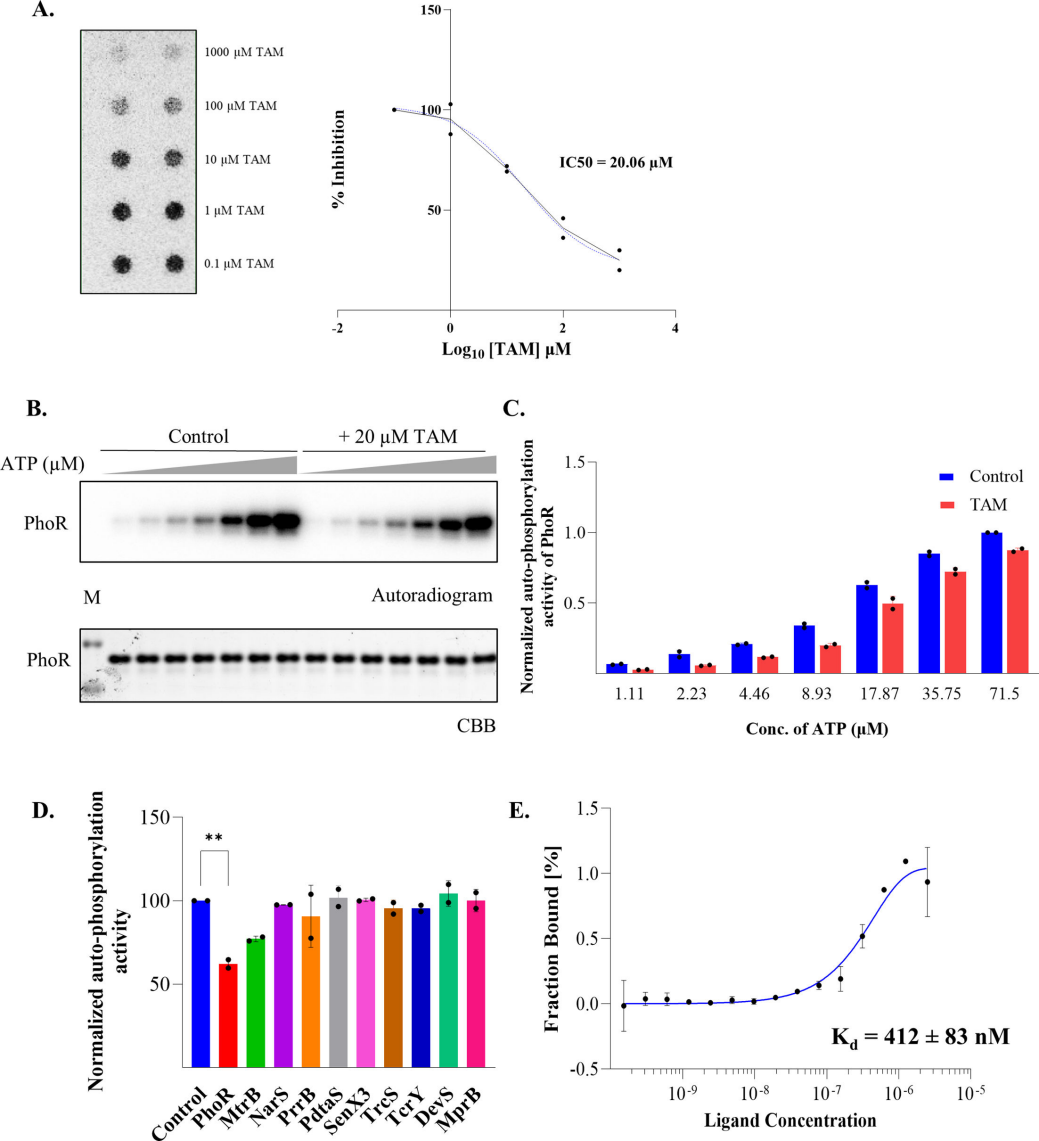
Characterization of TAM mechanism of action. (**A**) IC50 estimation of TAM-mediated inhibition of PhoR activity by dot blot with increasing concentration of TAM performed in duplicates. Densitometric analysis of the autoradiogram is shown on the *right*. (**B**) ATP competition assay. Kinase assay of PhoR was performed with DMSO (control) and TAM in the presence of increasing concentrations of (ɣ-^32^P) ATP, as indicated. The assay was set up in duplicate with various ATP concentrations for 60 min and visualized by autoradiography (top), and Coomassie Brilliant Blue (bottom) to check for equal protein loading. (**C**) Densitometric analysis of the autoradiogram in Fig. 4B is shown. Mean ± SD from two duplicate experiments is plotted. (**D**) Effect of TAM on the autophosphorylation activity of *M. tb* HKs (as indicated). ATP depletion assays were performed in duplicate, and normalized ATPase activities are plotted for the specified HKs (±TAM). Mean ± SD values are plotted as bar graphs. Data for every HK are presented with respect to minus drug control (set at 100%). ** denotes *P* < 0.01 for the difference in PhoR autophosphorylation activity in the presence and absence of TAM. (**E**) TAM binds MtrB HK with lower affinity. MST showing K_d_-Fit analysis of MtrB-GFP with TAM. The experiment was performed in triplicate, and a representative graph is shown.

To understand the mechanism of TAM action, we investigated its sensitivity and specificity and its competition with ATP. We used a dot blot assay investigating the effect of varying concentrations of TAM on the inhibition of PhoR autophosphorylation. Two independent experiments, each having two technical replicates, were performed (Fig. 4A). Densitometric analysis of all replicates was conducted to determine the IC50 value of TAM. As shown in Fig. 4A, the IC50 for TAM was estimated to be 20.06 µM.

Given the expansive overlap between the predicted TAM- and ATP-binding sites on PhoR at *Site 1* (Table S1), we anticipated a competition between them for binding to PhoR. A competition assay was performed with increasing concentrations of [ɣ-^32^P] ATP (range 1.0–72 μM) and 20 μM TAM (Fig. 4B). TAM inhibited PhoR autophosphorylation significantly when low concentrations of ATP were used. A gradual increase in ATP concentration resulted in a consequential decrease in the autophosphorylation-inhibiting action of TAM (Fig. 4B and C), highlighting a competition between TAM and ATP for binding to PhoR.

Typically, HKs display high conservation in their active sites (41), and since TAM competes with ATP to bind PhoR (Fig. 4B), thereby abrogating its autophosphorylation activity, we investigated whether TAM could inhibit other *M. tb* HKs as well. We purified the kinase domains of ten other mycobacterial HKs (Fig. S2) and performed an ATP depletion assay to check for the difference in the autokinase activity of HKs in the presence and absence of TAM. A decrease in the ATP concentration in the reaction is directly proportional to HK activity. As shown in Fig. 4D and Fig. S3, besides PhoR, one other HK, MtrB, displayed reduced activity in the presence of TAM; however, it was still considerably less than that of PhoR. To confirm this, we performed MST analysis for TAM binding to MtrB. We observed that TAM bound MtrB with a much higher dissociation constant K_d_ of 412 ± 83 nM (Fig. 4E), i.e., lower affinity than that noted with TAM-PhoR. Given that PhoR and MtrB share ~ 68.5% conservation in the TAM binding site residues (Fig. S4), the inhibition of MtrB ATPase activity by TAM is not surprising and, moreover, highlights the presence of other regulatory proteins /pathways as TAM targets. Overall, eleven *M. tb* HKs were tested in this study, and two were established as targets of TAM. Based on these data, we concluded that TAM shows strong binding to the PhoR HK and, more importantly, competes with ATP for PhoR binding and abolishes its autokinase activity, thereby having the potential to impact its downstream signaling.

### TAM affects *M. bovis* BCG growth and downstream PhoR-mediated signaling

**Fig 5:**
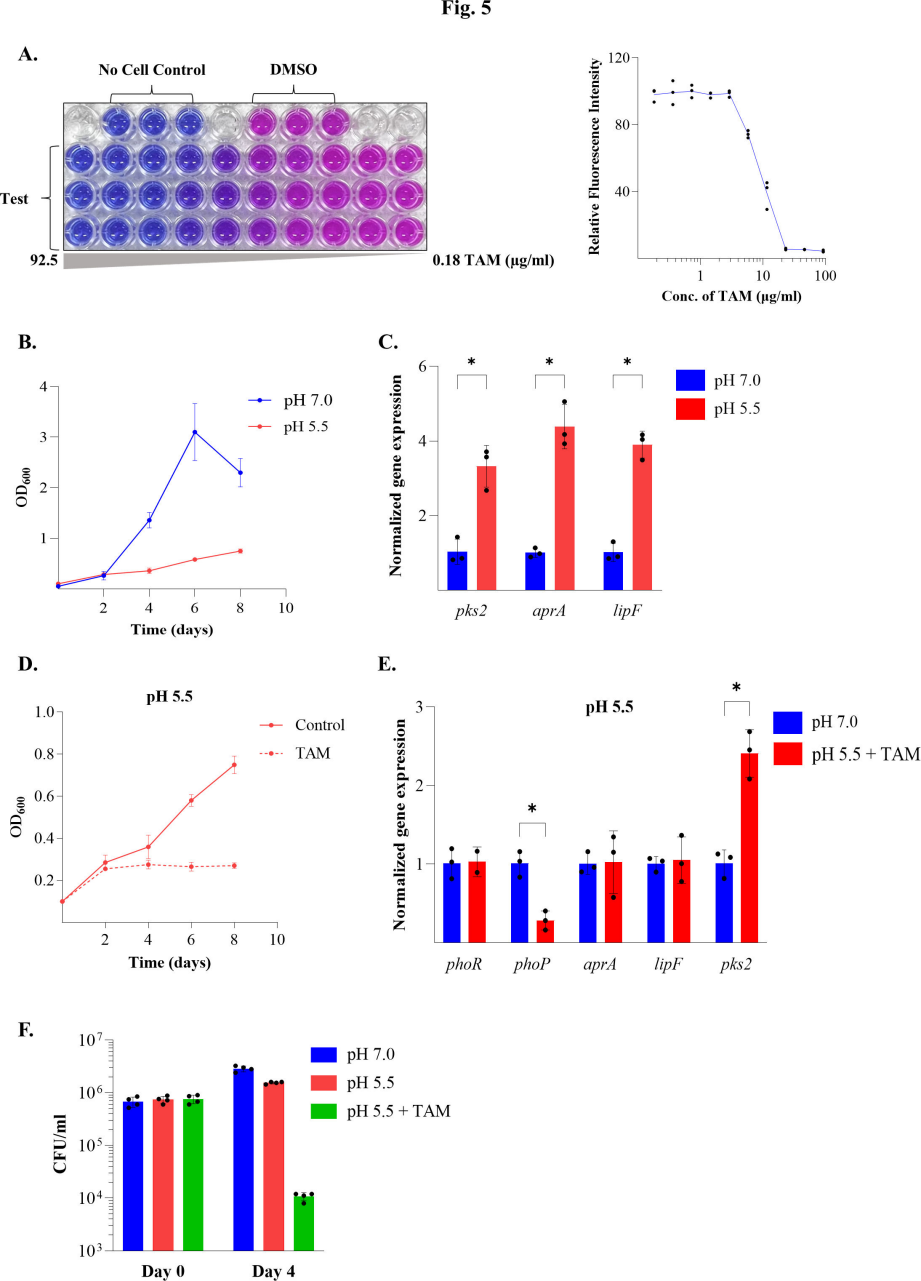
*In vivo* assessment of TAM on *M. bovis* BCG growth and signaling. (**A**) MIC determination of TAM with *M. bovis* BCG using a resazurin microtiter assay (REMA). (*Left*) 96-well plate containing *M. bovis* BCG exposed to different concentrations of TAM (0.18–92.5 μg/mL range), followed by incubation with 0.002% resazurin as described in Materials and Methods. The conversion of nonfluorescent, blue resazurin to fluorescent, pink resorufin indicates the presence of metabolically active cells. (*Right*) relative fluorescence values obtained after incubating the cells grown in a range of TAM concentrations. (**B**) Effect of different pH values on the growth of *M. bovis* BCG, plotted as OD_600_ versus time (days) at pH 7.0 and pH 5.5, and (**C**) acid-responsive gene expression at pH 7.0 and pH 5.5. Quantitative RT-PCR (qRT-PCR) analysis of acid-responsive genes *aprA, pks2,* and *lipF* was performed in RNA isolated from *M. bovis* BCG cultures grown at pH 5.5 relative to the expression observed at pH 7.0 (baseline set at 1.0). Data are presented from three independent replicates, and mean ± SD values are plotted as bar graphs. (**D**) Growth of *M. bovis* BCG in the absence (control) and presence of TAM, respectively, at pH 5.5. (**E**) Gene expression analysis by qRT-PCR of *phoPR* and its downstream target genes *aprA, lipF,* and *pks2* in *M. bovis* BCG grown at pH 5.5 in the presence of TAM. Data are presented from three independent replicates, and mean ± SD values are plotted as bar graphs. Fold change is reported with respect to vehicle control grown at pH 7.0 (baseline as 1.0). * denotes *P* < 0.05 for the differences observed in test conditions with respect to the control. (**F**) CFU analysis for the effect of TAM on the growth of *M. bovis* BCG at pH 5.5. Bar graphs represent the number of colonies observed at day 0 and at day 4 at pH 7.0 and pH 5.5 (in the presence and absence of TAM). Data are presented from two independent experiments, each performed as two biological replicates (*N* = 2, *n* = 2).

Major frontline anti-tubercular drugs like isoniazid, ethambutol, and rifampicin have minimum inhibitory concentration (MIC) values of 0.25–10 μg/mL for different *M. tb* strains (42), which inhibit or slow down the growth of drug-resistant strains of mycobacteria. Previously, the MIC for TAM has been reported in the range of 6.75 μg/mL to 12 μg/mL (43). To account for any variation between studies, we determined the MIC50 for TAM in our experiments and found it to be in the range of 5.78 μg/mL to 11.5 μg/mL (Fig. 5A). Based on these results, we used a sublethal concentration of TAM (10 μg/mL) to investigate its effect on the growth of *M. bovis* BCG.

Given that activation of PhoR is proposed to occur at low pH (16, 18, 19), we conducted growth experiments at acidic pH (pH 5.5) and neutral pH (pH 7.0). Under this experimental setup, the cells grew poorly in acidic pH (Fig. 5B). We isolated RNA from these cultures and analyzed the expression of PhoPR-dependent, acid-responsive genes such as *aprA* (16), *pks2* (23), and *lipF* (23). As shown in Fig. 5C, the transcripts for *aprA* (~4.38 fold), *pks2* (~3.3 fold), and *lipF* (~3.9 fold) were significantly upregulated under acidic pH relative to the pH 7.0 control. These results validated our experimental conditions, indicating PhoPR activation. Next, we followed the growth of *M. bovis* BCG under acidic pH, with DMSO (control) or TAM inhibitor. As shown in Fig. 5D, the growth was significantly impacted in the presence of TAM at pH 5.5, with ~50% reduction in the optical density. qRT-PCR analysis of RNA isolated from cells subjected to growth under acidic conditions in the presence of TAM revealed loss of upregulation of all target genes, except *pks2* (Fig. 5E), observed earlier in pH 5.5 (Fig. 5C). In fact, *phoP* transcripts were found to be significantly downregulated in the presence of TAM, possibly due to PhoR inhibition and subsequent disruption of phosphotransfer to PhoP. Since *phoP* RR expression is known to be under autoregulatory control (17), lower levels of activated PhoP~*P* are expected to reduce the expression of *phoPR* genes. The continued upregulation of *pks2* expression under acidic conditions in the presence of TAM suggests PhoR-independent regulatory effects. To confirm whether the effect of TAM on *M. bovis* BCG is bacteriostatic or bactericidal, we estimated viable counts under pH 7.0 and pH 5.5 (± TAM) (Fig. 5F). We noted a significant decrease in the number of viable counts post-TAM treatment (Fig. 5F). These data not only validate earlier observations (Fig. 5B and D) but also indicate a bactericidal effect of TAM on *M. bovis* BCG under acidic conditions. Collectively, these results confirm the antimicrobial activity of TAM and suggest a plausible mechanism for TAM action in mycobacteria. Given that the observed effect suppresses adaptation to low pH, TAM emerges as a potent antimycobacterial agent that facilitates the elimination of bacteria by increasing their susceptibility to acidic conditions, thereby disrupting one of the key processes needed for intracellular survival of *M. tb*.

## DISCUSSION

Two-component signaling proteins are desirable drug target candidates because they drive adaptive responses to extracellular or intracellular cues, including antimicrobial resistance, regulation of virulence, maintenance of bacterial growth, and intracellular survival (41). Over the years, several TCSS inhibitors with diverse chemical and structural properties have been identified and characterized (41). While both the sensor kinase and the DNA-binding RR are druggable targets, there is an inherent benefit of shutting down a signaling pathway by inhibiting the HK action as the cascade can be inhibited at the first step. Moreover, the conserved features of HK domains may facilitate the development of inhibitors possessing broad-spectrum antimicrobial activity.

In this study, we focused on evaluating the potential of *M. tb* PhoR HK as a novel antimycobacterial drug target primarily due to its role in virulence (21), regulation of essential cell wall lipid synthesis (23, 30), and its complex network with various signaling proteins, including TCSSs (29, 32, 34) and Ser/Thr protein kinases (35). The outcomes of this study were multipronged and included (i) developing and optimizing an autokinase activity-based HTS assay for PhoR HK; (ii) screening for small-molecule inhibitors that abolish PhoR autophosphorylation; and (iii) characterization of the mechanism of action of the inhibitor using structural and functional interrogation of inhibitor-PhoR interactions on mycobacterial growth and downstream regulation of gene expression.

HKs, being multifunctional enzymes, can autophosphorylate, phosphotransfer, and dephosphorylate their cognate RR (or other noncognate RRs/substrate proteins) *(reviewed in* [41]). All three enzymatic activities are possible points for targeting inhibition. Several studies have identified HK inhibitors and investigated their suitability for anti-TB therapy *(reviewed in* [41]). Except for inhibitor molecules like HC102A, HC103A, and BTP15, which inhibit the autokinase activity of DevS/DosT (44) and MprB histidine kinases (45), respectively, the direct inhibition of PhoR function has not been investigated. In a study, ethoxzolamide (ETZ) has been proposed to indirectly inhibit the PhoR sensing mechanism by targeting cell surface carbonic anhydrases that may modulate the local extracellular environment (46); however, no experimental evidence supports that it inhibits PhoR autophosphorylation. Recently, Watson et al. reported that ETZ lowered the expression of the target gene *aprA* and reduced CFU in a cell line infection model (17); however, no mechanism for ETZ action was proposed.

In our screening exercise for PhoR autokinase inhibitors, after applying cut-off value filters based on drug pharmacology, toxicity, and false lead filtration, only two hits were obtained, coincidentally for the same compound, TAM. It was identified from two different compound libraries: plate 4888 of the mechanistic set and plate 4891 of the oncology set of the NIH library, thereby raising the confidence of our data. TAM is a selective estrogen receptor modulator (SERM) that has proven very effective in the treatment of breast cancer (36, 47). Having antimicrobial activity against many microbes, SERMs have the potential to be repurposed to control bacterial infections (43, 48–50). Remarkably, TAM and its metabolites, including N-desmethyltamoxifen, 4-hydroxytamoxifen, and endoxifen, have shown potent antibacterial activity against methicillin-resistant *Staphylococcus epidermidis* and vancomycin-resistant *Enterococcus faecalis* (51). These metabolites also reduced bacterial loads and increased survival rates in mice infected with gram-negative bacteria such as *Acinetobacter baumannii* and *Escherichia coli* (52). In a recent study, Boland et al. have shown that TAM exhibits antimycobacterial activity against both drug-sensitive and drug-resistant *M. tb* strains (49). It effectively reduces the number of intracellular tubercular bacilli in macrophages in a dose-dependent manner (49). Despite these documented studies, the underlying mechanisms of pathogen-directed effects of TAM remain unclear. Because TAM is known to enhance neutrophil innate immune function and modulate other host pathways, such as autophagy and lysosome function, its potential to act as a host-directed therapeutic for TB holds promise. Published data support the notion that TAM’s increase in lysosomal activation equips the host to control intracellular replication of pathogens such as *M. tb* (43, 50).

To the best of our knowledge, no mycobacterial target for TAM has yet been reported. We demonstrate that TAM competes with ATP to bind PhoR HK with high affinity and abrogates PhoR autophosphorylation. Given the fact that PhoPR TCS is responsible for mycobacterial adaptation to acidic pH within the macrophages (19) and is necessary for intracellular survival (18, 19), we propose that TAM-mediated inhibition of PhoR activation disrupts the downstream PhoPR signaling, leading to decreased growth of the bacteria in acidic conditions. Because TAM is involved in regulating the host’s lipid biosynthesis pathway (53) and has inhibitory activity on the *M. tb* PhoR kinase (Fig. 2), a key determinant in mycobacterial membrane remodeling, we hypothesize that TAM may modulate lipid biosynthesis, affecting membrane architecture, permeability, and overall fitness of mycobacteria. Owing to its antimycobacterial activity, favorable pharmacokinetic profile, and FDA-approved clinical use in cancer treatment, TAM has been repurposed in TB control, offering an attractive solution, avoiding the long drug-discovery pipeline. However, the challenges to implementing TAM in the antitubercular treatment require extensive investigations to fully comprehend the host-independent, TAM-mediated pathogen-directed effects.

*M. tuberculosis* contains multiple TCSSs that frequently interact with one another in complex networks. The PhoPR system exemplifies this complexity and connects with several atypical signaling pathways (31, 32, 34, 35). While our study identified PhoR as a direct target of TAM, other pathways and targets are likely involved. Since we used only the autophosphorylation-proficient sensor kinase domain for our screening, the probability of identifying a generalized inhibitor is very high. In agreement with this hypothesis, we found that TAM also moderately inhibited one another HK, MtrB, which is vital for regulating cell division (54), cell wall permeability (55), and its composition (56, 57), further confirming the compound’s broad effects. These findings highlight TAM’s pleiotropic effects on mycobacterial systems. Future research must aim to comprehensively examine TAM’s global impact on mycobacterial gene expression and proteomic profiles.

In the complex intracellular environment where multiple signaling networks interact, TCS inhibition may not directly kill the bacteria. However, it is likely to significantly reduce the pathogen’s fitness and survival capacity. This reduction in bacterial fitness could shift the balance in favor of the host immune system, potentially leading to enhanced lysosomal activation and subsequent intracellular bacterial elimination. Rather than requiring bactericidal effects, this approach leverages the host’s natural defense mechanisms to clear the infection. Our findings establish HKs such as PhoR as promising novel drug targets and demonstrate that TAM-mediated inhibition of PhoR HK represents a valuable step toward developing improved therapeutic strategies for TB treatment and eradication. This research opens new avenues for combination therapies that could enhance current TB treatment regimens.

## MATERIALS AND METHODS

### Strains, plasmids, and chemicals

The strains and plasmids used in this study are listed in Table S2. Luria Bertani (LB) broth and/or LB Agar were used to grow all the strains of *E. coli* at 37°C. For large-scale protein purification, 2× YT was used to cultivate *E. coli* BL21 (DE3) cells. *M. bovis* BCG cultures were grown in Middlebrook 7H9 liquid media and 7H11 solid media supplemented with 5% albumin, 2% dextrose, and 0.85% sodium chloride and 0.05% oleic acid, 5% albumin, 2% dextrose, and 0.85% sodium chloride supplement, respectively, and 0.05% Tween 80 at 37°C with aeration at 180 rpm. TAM (S1238) was procured from SelleckChem, USA. Other routine chemicals like ampicillin (Amp; 100 µg/mL), isopropyl-β-D-thiogalactopyranoside (IPTG; 1 mM), DMSO (vol/vol), and imidazole (250 mM) were obtained from Merck, unless specified.

### Protein expression and purification

All recombinant proteins mentioned in Table S2 were overexpressed and purified from *E. coli* BL21 (DE3) using standard procedures. Briefly, a single colony was inoculated in 5 mL of LB broth and cultured overnight at 37°C with shaking at 180 rpm. A volume of 400 mL of 2× YT broth was then inoculated with 4 mL of overnight-grown culture and grown at 37°C with shaking till the OD_600_ reached 0.4–0.6. The culture was induced with 1.0 mM IPTG at 16°C for 16–18 h. Subsequently, cells were harvested by centrifugation at 3,500 rpm for 10 min at 4°C. The cell pellet was resuspended in Lysis Buffer (50 mM Tris pH 8.0, 300 mM NaCl, 10 mM imidazole, and protease inhibitor cocktail) and lysed by sonication (20 min, pulses of 2 s ON and 2 s OFF, 35% amplitude). The soluble fraction was separated by centrifugation of the lysate at 13,000 rpm for 30 min at 4°C. The supernatant was filtered using a 0.2 μM filter and applied to the His-Trap FF column pre-equilibrated with lysis buffer. His-tagged HKs were purified using Ni-NTA affinity chromatography. For eluting the proteins, an elution buffer (50 mM Tris pH 8.0, 300 mM NaCl, 250 mM imidazole) was used, and 1 mL fractions were collected. The fractions were pooled and dialyzed to remove excess salt and imidazole, followed by SDS-PAGE analysis and protein estimation using Bradford’s assay.

### *In vitro* kinase assay

For radioactive kinase assays, 90–100 pmoles of PhoR (or other HKs as indicated) with DMSO or TAM was taken in a 20 μL reaction with 1× kinase buffer (50 mM Tris pH 8.0, 20 mM MgCl2, and 1.0 mM DTT) for 30 min at 30°C. Subsequently, 50 μCi (ɣ-^32^P)-labeled ATP was added to check for autophosphorylation activity for 1 h at 30°C. The reactions were stopped by the addition of 5× SDS loading dye. The samples were resolved on 12% SDS-PAGE gels and analyzed using autoradiography.

For nonradioactive kinase assays, an ATP depletion assay was standardized as per the manufacturer’s instructions (ATP Determination Kit, Invitrogen). Briefly, 100 pmoles of HKs was incubated with DMSO (control) or TAM for 30 min at room temperature (RT) in the presence of 1× kinase Buffer. Then, 5 μM ATP was added, and the reaction mix was incubated at 30°C for 30 min, followed by the addition of Luciferin-Luciferase mix. Luminescence was recorded using a multimode microplate reader, and mean ± SD values are reported.

### HTS assay to screen for small-molecule inhibitors

To screen for compounds inhibiting autophosphorylation of PhoR, a 96-well plate assay using a 96-well Dot Blot (Bio-Dot, Bio-Rad Laboratories) was devised. One microgram of PhoR-GFP was added to 1 mL of 1× kinase buffer and divided into 96-well plates containing 10 μM of different compounds each and incubated at RT for 30 min. Ten microliters of 1× kinase buffer containing 1 μCi (ɣ-^32^P) labeled ATP was then added to each well containing PhoR-GFP with compounds and incubated at 30°C for 1 h. Reaction mixtures were then transferred onto a nitrocellulose membrane using the dot blot apparatus. The membrane was then washed with 1× PBS to remove unbound ATP and imaged for GFP fluorescence to check for equal loading of the proteins before analyzing for autophosphorylation using autoradiography. The intensity of the spots from the autoradiograms was normalized with the fluorescent readings of the respective spots and plotted as scatter plots.

For IC50 calculations using Dot-blots, data were analyzed using the nonlinear fit function of the GraphPad Prism software (Version 10.4.1) for log (inhibitor) vs response—Variable slope (four parameters) with X as the concentration of log_10_ [TAM] in micromolars.

### Z-score calculation

To calculate the Z-score of the assay, PhoR-GFP incubated with DMSO and PhoR-GFP with EDTA were used as PC and NC, respectively. One half of a 96-well plate was loaded with PC and the other half with NC. 1× kinase buffer containing 1 μCi (ɣ-^32^P) labeled ATP was then added to each well and incubated at 30°C for 1 h. Reaction mixtures were transferred onto the nitrocellulose membrane and analyzed as described above. Z-score was calculated using standard deviations and variances using the formula, *Z-score = 1-[{3*(*σPC+*σ*NC*)*}/(|µPC*−*µNC|)]*.

### MST

To determine the binding affinity of TAM with PhoR or MtrB, TAM was dissolved in DMSO (10 mM), and serial dilutions of TAM were made in 16 different capillaries containing 50 μM PhoR-GFP or MtrB-GFP (58) such that the fluorescence in the NanoTemper Monolith is in the range of 1,200–1,400 U. The capillaries containing the reaction mix were then subjected to MST analysis. PhoR-GFP/MtrB-GFP and DMSO were used as controls. Data were analyzed using MO Affinity analysis software (NanoTemper Technologies).

### PhoR structure retrieval, validation, and molecular docking

Due to the lack of availability of a crystal structure of PhoR, a computationally predicted structure of PhoR was obtained from the AlphaFold database (AF-P71815-F1). The structure was also validated using PROCHECK (37), which provides information about Ramachandran plot statistics, backbone conformation, stereochemical quality, and the ERRAT program, which examines non-bound atomic interactions in protein models (38).

Molecular docking was carried out for the kinase domain region of PhoR (256–470 aa) with TAM or ATP, as indicated. Prior to docking, the target protein was prepared using Protein Preparation Wizard (Schrodinger, LLC, New York, NY), which involved the addition of the hydrogen atoms, assigning of bond orders, formation of disulfide bonds, and removal of hetero-atoms, followed by energy minimization and refinement. The ligand structure was prepared using the LigPrep module, and the binding site was determined using the SiteMap module (39). Subsequently, molecular docking was done using the XP mode of Glide (40), and the ΔG bind for each docked pose was further estimated using the Prime MM-GBSA method (59). The docked complex structure of PhoR-TAM was used to further dock the phosphoryl group at the His^259^ residue.

### MIC determination

MIC was determined as described by Shee et al (60). Briefly, a REMA with sterile 96-well flat-bottom plates was used. *M. bovis* BCG was cultured in 7H9+ADS medium and grown till exponential phase (OD_600_ ~ 0.4 to 0.8). Approximately 10^5^ bacteria (corresponding to OD_600_ ~ 0.05) per well were added in a total volume of 200 μL of 7H9+ADS medium. Wells lacking *M. bovis* BCG served as no cell control. Additional control consisted of wells containing cells without drug treatment (DMSO). After 4 days of incubation at 37°C in the presence of various concentrations of TAM, 20 μL of 0.02% resazurin was added, and plates were incubated for an additional 24 h. Fluorescence intensity was measured with excitation at 530 nm and emission at 590 nm. The MIC50 was taken as the lowest drug concentration that resulted in at least a 50% reduction in fluorescence compared to the untreated growth control.

### Phenotypic growth assay

*M. bovis* BCG cultures were grown in 7H9+ADS medium and 0.05% Tween 80 till the log phase. The cells were then transferred to pH-adjusted media (pH 5.5 and pH 7.0), at a starting OD_600_ of 0.05, and growth was followed for 8 days. To check the effect of TAM on growth, DMSO (control) or TAM (10 μg/mL) was added to cultures containing cells with a starting OD_600_ of 0.05 in pH-adjusted media with pH 5.5, and OD_600_ measurements were taken as described above. For RNA isolation and Real-Time RT-PCR experiments, *M. bovis* BCG cultures were grown for 4–6 days (OD_600_ ~2.0). The cells were then pelleted down, washed with PBS, and resuspended in pH-adjusted media (pH 5.5) at a final OD_600_ of 0.025 and allowed to grow for 4 days (OD_600_ ~0.8) before exposure to TAM for 72 h. The cells were pelleted, washed with PBS, and stored at −70°C for RNA isolation.

### RNA isolation

For gene expression analysis, mycobacterial culture pellets were processed for RNA isolation using the RNeasy Mini Kit as per the manufacturer’s protocol. Briefly, the cells were resuspended in RLT buffer and lysed with 500 μL of sterile zirconium beads using a bead beater in 6 cycles of 30 sec at 30 Hz frequency. Beads were separated by centrifugation at 12,000 rpm for 5 min at 4°C, and the supernatant was transferred to fresh 1.5 mL microcentrifuge tubes, and 1 volume of 70% ethanol was added. Seven-hundred microliters of the sample was transferred to the RNeasy Mini spin columns and centrifuged, followed by washing with RW1 and RPE buffers. The columns were placed in fresh 1.5 mL microcentrifuge tubes, and RNA was eluted with nuclease-free water. DNase treatment was done using the Turbo DNA-free Kit as per the manufacturer’s protocol.

### Real-time RT-PCR

qRT-PCR analysis was done for selected genes using gene-specific primers as described (17) except *lipF,* for which the primers used are forward primer- 5′ GTGGTGCTCTATTTGCACGG 3′ and reverse primer- 5′ ATCCCCAGCGAATGCTTAGG 3′. Briefly, cDNA was synthesized using random hexamers supplied in the Verso cDNA synthesis kit (Thermo Scientific). cDNA (100 ng) prepared from RNA isolated from vehicle control and TAM-grown cells was aliquoted into different wells of a 96-well plate, and PCR Master mix (PowerUp SYBR Green Master Mix, Applied Biosystems) was added to each well along with the specific primer pair for each gene. The reactions were then subjected to 35 cycles of qPCR, and threshold cycle (C_t_) values were calculated for each gene. The calculated C_t_ value for each gene was normalized to the *sigA* gene (internal control), followed by that of the gene of interest in the vehicle control cells to determine the fold change. Data from three independent biological replicates were analyzed and reported.

### CFU determination

*M. bovis* BCG cultures were grown in 7H9+ADS medium and 0.05% Tween 80 till the log phase. The cells were then transferred to pH-adjusted media (pH 5.5 and pH 7.0) at a starting OD_600_ of 0.05. To check the effect of TAM on growth, DMSO (control) or TAM (10 μg/mL) was added to cultures in pH-adjusted media. Viable counts were estimated by plating cultures on 7H11+OADS agar plates at specified time points.

### Statistical analysis

Statistical analysis was done using multiple unpaired *t*-tests (Prism 10.4.1 software) for qRT-PCR experiments with the variance assumption as individual variance for each row. Unpaired Student’s *t*-tests were done for all the kinase assays (radioactive and nonradioactive ATP depletion assays). A *P* value of <0.05 was considered significant.

## Data Availability

The data presented are available within the article and its supplemental material.

## EDITOR'S NOTE

**Ed. Note**: A potential conflict of interest was identified post-acceptance, and the senior editor conducted an additional review to ensure the integrity and fairness of the peer review process.

## SUPPLEMENTAL MATERIAL

Supplemental tables and figures spectrum.01880-25-s0001.pdf Tables S1 and S2, and Figures S1 to S4.

ASM does not own the copyrights to Supplemental Material that may be linked to, or accessed through, an article. The authors have granted ASM a non-exclusive, world-wide license to publish the Supplemental Material files. Please contact the corresponding author directly for reuse.
