# Supplementary material for: Tamoxifen inhibits histidine kinases of M. tuberculosis two-component signaling systems
Source: Microbiol Spectr. 2025 Dec 16;14(2):e01880-25. doi: 10.1128/spectrum.01880-25 (PMC12889096; doi:10.1128/spectrum.01880-25)
Supplement: Supplemental tables and figures — Tables S1 and S2, and Figures S1 to S4. [file spectrum.01880-25-s0001.pdf]

Table S1: PhoR residues interacting with TAM and ATP revealed by Molecular Docking.

PhoR-Tamoxifen, -52.68 kcal/mol

**Leu**	**299**
Leu	300
**Asp**	**303**
Gln	365
Arg	368
**Asn**	**369**
**Ala**	**372**
**Asn**	**373**
**Gln**	**376**
**His**	**377**
**Val**	**414**
**Phe**	**418**
**Tyr**	**419**
**Arg**	**420**
**Ala**	**421**
Ser	429
**Gly**	**430**
**Gly**	**431**
**Thr**	**432**
**Gly**	**433**
**Leu**	**434**
**Gly**	**435**
**Leu**	**436**
Ser	437
**Ile**	**438**

PhoR-ATP, -20.71kcal/mol

**Leu**	**299**
**Asp**	**303**
**Arg**	**368**
**Asn**	**369**
**Ala**	**372**
**Asn**	**373**
**Gln**	**376**
**His**	**377**
Thr	378
Asp	401
Pro	404
Gly	405
Met	406
**Val**	**414**
Glu	416
**Phe**	**418**
**Tyr**	**419**
**Arg**	**420**
**Ala**	**421**
Asp	422
**Gly**	**430**
**Gly**	**431**
**Thr**	**432**
**Gly**	**433**
**Leu**	**434**
**Gly**	**435**
**Leu**	**436**
**Ile**	**438**
Cys	460
Phe	462

Bold entries represent common interacting residues of PhoR with TAM and ATP.

Table S2. List of Strains and Plasmids used in the study.

Strain or plasmid construct	Description	Source or Reference
E. coli DH5α	Δ*(argF-lac)169*, φ80d*lacZ58*(M15), Δ*phoA8*, *glnX44*(AS), *deoR481*, *rfbC1*, *gyrA96*(NalR), *recA1*, *endA1*, *thiE1* and *hsdR17*	Lab collection
E. coli BL21 (DE3)	F^- *ompT gal dcm lon* $hsdS_B(r_B^- m_B^-)$ λ(DE3 [*lacI lacUV5-T7p07 ind1 sam7 nin5*]) $[malB^+]_{K-12}(\lambda^S)$	Lab Collection
M. bovis BCG	*Mycobacterium bovis* BCG strain TMC 1011 isolated from bovine milk	Lab Collection
pProEx-HTa	Expression vector with N-terminal 6xHis-Tag, Amp^r	Invitrogen Inc., USA
pProEx-HTa::*phoR*	Catalytic C-terminal domain of *phoR* (684 -1458 bp) cloned in pProEx-Hta with N-terminal 6xHis-Tag, Amp^r	(34)
pProEx-HTc::*mtrB*	Catalytic C-terminal domain of *mtrB* (851 -1754 bp) cloned in pProEx-Htc with N-terminal 6xHis-Tag, Amp^r	(34)
pProEx-HTa::*narS*	Catalytic C-terminal domain of *narS* (657 -1278 bp) cloned in pProEx-Hta with N-terminal 6xHis-Tag, Amp^r	(34)
pProEx-HTa::*prrB*	Catalytic C-terminal domain of *prrB* (670 -1391bp) cloned in pProEx-Hta with N-terminal 6xHis-Tag, Amp^r	(34)
pProEx-HTb::*pdtaS*	Catalytic C-terminal domain of *pdtaS* (817 -1506 bp) cloned in pProEx-Htb with N-terminal 6xHis-Tag, Amp^r	(34)
pProEx-HTa::*senX3*	Catalytic C-terminal domain of *senX3* (427 -1233 bp) cloned in pProEx-Hta with N-terminal 6xHis-Tag, Amp^r	(34)
pProEx-HTb::*trcS*	Catalytic C-terminal domain of *trcS* (751 -1530 bp) cloned in pProEx-Htb with N-terminal 6xHis-Tag, Amp^r	(34)
pProEx-HTc::*tcrY*	Catalytic C-terminal domain of *tcrY* (721 -1428 bp) cloned in pProEx-Htc with N-terminal 6xHis-Tag, Amp^r	(34)
pProEx-HTc::*devS*	Catalytic C-terminal domain of *devS* (1134 -1734 bp) cloned in pProEx-Htc with N-terminal 6xHis-Tag, Amp^r	(12)
pProEx-HTc::*mprB*	Catalytic C-terminal domain of *mprB* (631 -1515 bp) cloned in pProEx-Htc with N-terminal 6xHis-Tag, Amp^r	(34)
pProEx-HTb-GFP::*phoR*	Catalytic C-terminal domain of *phoR* (684 - 1458 bp) fused to GFP with N-terminal 6xHis-Tag, Amp^r	(58)
pProEx-HTb-GFP::*mtrB*	Catalytic C-terminal domain of *mtrB* (851 - 1754 bp) fused to GFP with N-terminal 6xHis-Tag, Amp^r	(58)

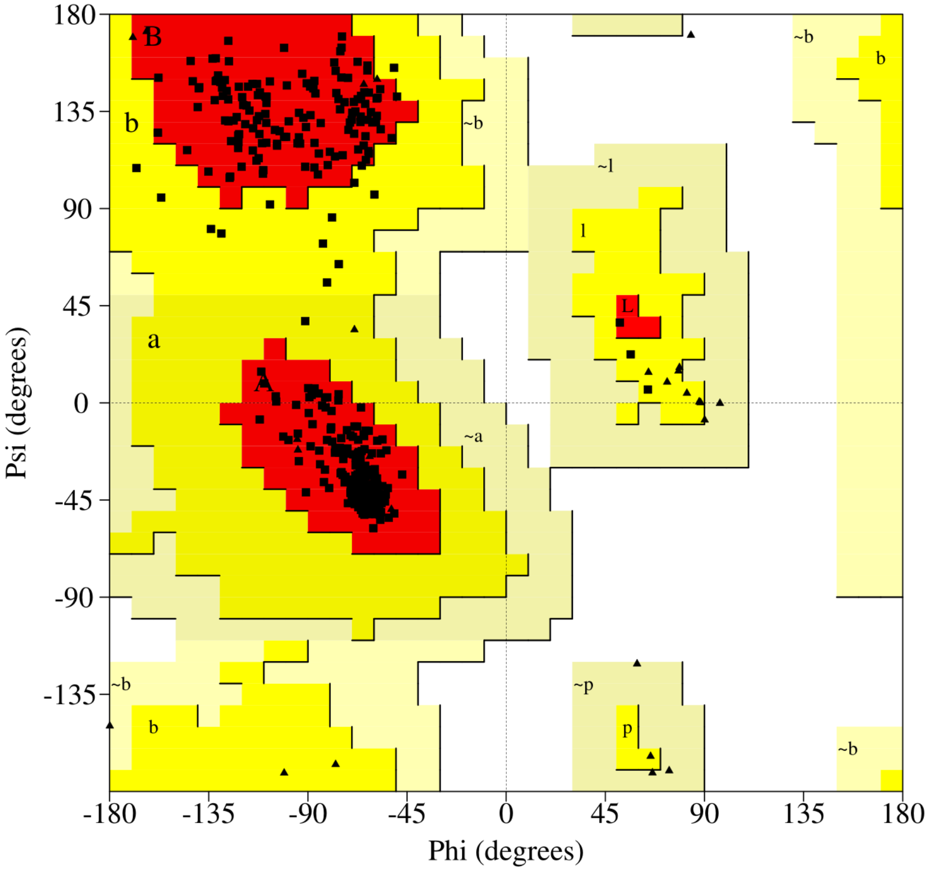

Fig. S1. Ramachandran Plot for structural validation of PhoR.

Structural validation of PhoR, revealing the 485 amino acid predicted structure with 96.7% residues in the favourable region.

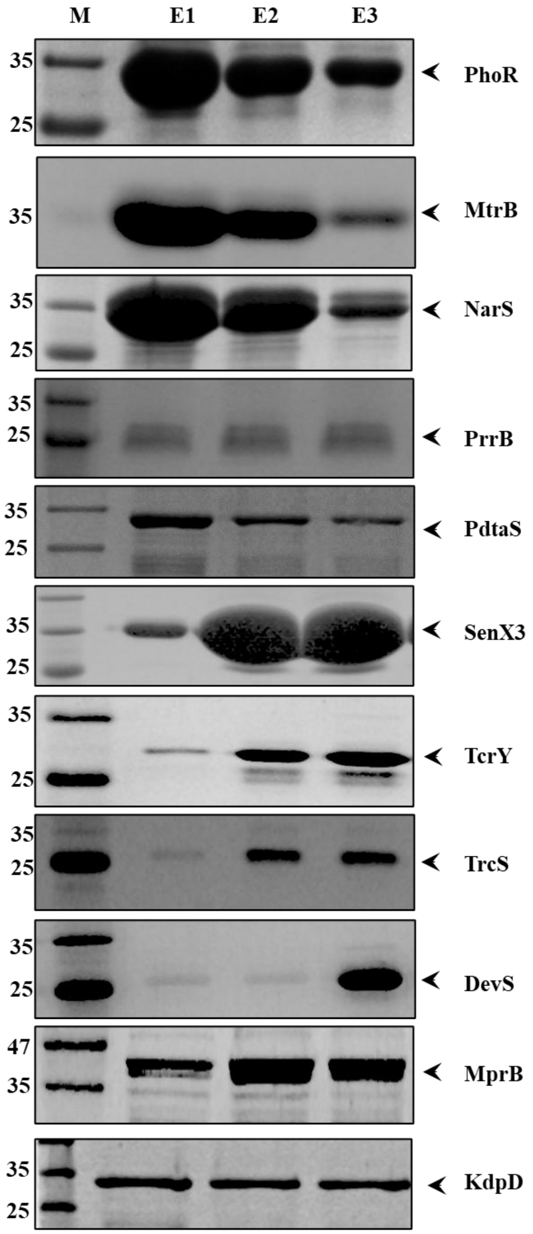

Fig. S2. Purification of Kinase Domain of HKs using Affinity Chromatography.

Recombinant His-tagged Kinase Domains of HKs (n = 11) were purified from *E. coli* BL21(DE3) using Ni-NTA affinity chromatography. SDS PAGE analysis of the eluates (E) is shown. Molecular weight Marker (M) denotes molecular sizes in kDa.

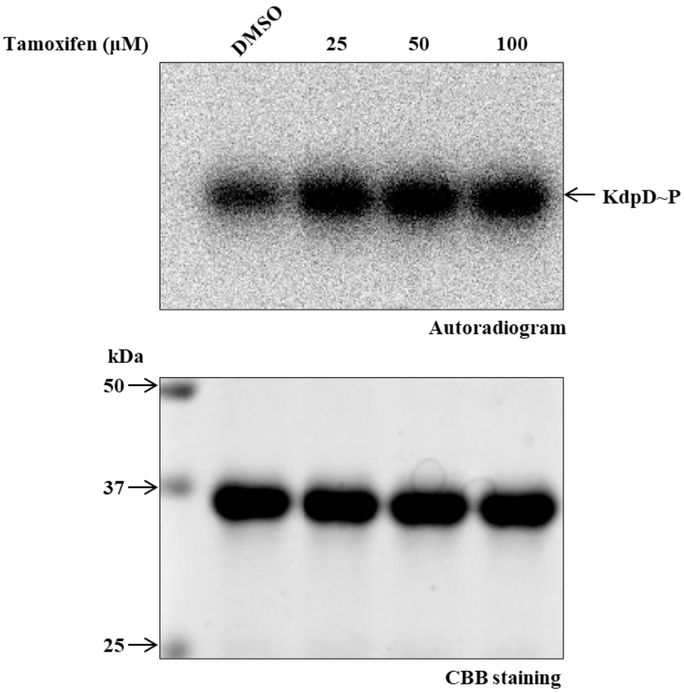

Fig. S3. Effect of Tamoxifen on Autophosphorylation of KdpD.

Recombinant His-tagged Kinase Domain of KdpD were purified from *E. coli* using Ni-NTA affinity chromatography, and the radioactive kinase assay was performed in the presence and absence of TAM at various concentrations.

A.

PhoR Interacting residues (35 total in Site 1; *256-445*): *258, 259, 260, 261, 262, 263, 264, 266, 267, 293, 296, 297, 300, 330, 333, 334, 337, 345, 361,364, 365, 366, 368, 369, 371, 372, 373, 375, 376, 420, 425, 430, 431, 434, 435*

```
PhoR      ---------------DASHELRTPLTTIRGFAELY--RQGAA---RDVG-----MLLSRI   35
MtrB      ---------------DVSHELRTPLTTVRMAADLI--YDHSADLDPTLR-----RSTELM   38
:

PhoR      ESEASRMGLLVDDLLLLARLDAHRP-LELCRVDLLALASDAAHDA-RAMDPK--RRITLE   91
MtrB      VSELDRFETLLNDLLEISRHDAGVAELSVEAVDLRTTVNNALGNV-GHLAEEAGIELLVD   97
.

PhoR      VLDGPGTPEVLGDESRLRQVLRNLVANAIQHTPESAD----VTVRVG-----TEGDDAIL   142
MtrB      LPAEQ--VIAEVDARRVERILRNLIANAIDHAEHK-P----VRIRMA-----ADEDTVAV   145

PhoR      EVADDGPGMSQEDALRVFERFYRADSSRARASGGTGLGLSIVDSLVAA-HGGAVTVTTAL   201
MtrB      TVRDYGVGLRPGEEKLVFSRFWRSDPSRVRRSGGTGLGLAISVEDARL-HQGRLEAWGEP   204

PhoR      G-EGCC----FRVSLPRVS       215
MtrB      G-EGAC----FRLTLPMVR       218
```

B.

Conservation of TAM interacting residues of PhoR with other HKs:

HK	No of Conserved Residues	Percentage (%)	Inhibition by TAM
**MtrB**	24	68.5	Yes
**PrrB**	22	62.8	No
**MprB**	15	42.8	No
**NarS**	6	17.1	No
**SenX3**	18	51.4	No
**PdtaS**	7	20	No
**DevS**	6	17.1	No
**DosT**	5	14.2	No
**TrcS**	22	62.8	No
**TcrY**	24	68.5	No
**KdpD**	15	42.8	No

Fig. S4. Conservation of residues of HKs interacting with TAM in PhoR.

(A) Sequence alignment of TAM-interacting residues of PhoR with other HKs. **(B)** Table representing the percentage conservation with PhoR and if inhibition was observed with TAM or not.
